# High‐intensity interval training and essential amino acid supplementation: Effects on muscle characteristics and whole‐body protein turnover

**DOI:** 10.14814/phy2.14655

**Published:** 2020-12-28

**Authors:** Katie R. Hirsch, Casey E. Greenwalt, Hannah E. Saylor, Lacey M. Gould, Courtney H. Harrison, Gabrielle J. Brewer, Malia N. M. Blue, Arny A. Ferrando, Kim M. Huffman, Elizabeth J. Mayer‐Davis, Eric D. Ryan, Abbie E. Smith‐Ryan

**Affiliations:** ^1^ Applied Physiology Laboratory Department of Exercise and Sport Science University of North Carolina at Chapel Hill Chapel Hill NC USA; ^2^ Human Movement Science Curriculum Department of Allied Health Science University of North Carolina at Chapel Hill Chapel Hill NC USA; ^3^ Department of Geriatrics Donald W. Reynolds Institute on Aging Center for Translational Research in Aging & Longevity University of Arkansas for Medical Sciences Little Rock AR USA; ^4^ Duke Molecular Physiology Institute Duke University Durham NC USA; ^5^ Department of Medicine Duke University School of Medicine Durham NC USA; ^6^ Department of Nutrition Gillings School of Public Health University of North Carolina at Chapel Hill Chapel Hill NC USA; ^7^ Department of Medicine University of North Carolina Chapel Hill NC USA; ^8^ Neuromuscular Assessment Laboratory Department of Exercise and Sport Science University of North Carolina at Chapel Hill Chapel Hill NC USA

**Keywords:** Exercise, Nutrition, Protein, Sex Differences, Ultrasound, Whole‐Body Protein Kinetics

## Abstract

The purpose of this study was to compare the independent and combined effects of high‐intensity interval training (HIIT) and essential amino acids (EAA) on lean mass, muscle characteristics of the quadriceps, and 24‐hr whole‐body protein turnover (WBPT) in overweight and obese adults. An exploratory aim was to evaluate potential modulatory effects of sex. Sixty‐six adults (50% female; Age: 36.7 ± 6.0 yrs; %BF: 36.0 ± 7.8%) were assigned to 8 wks of: (a) HIIT, 2 days/wk; (b) EAA supplementation, 3.6 g twice daily; (c) HIIT + EAA; or (d) control. At baseline, 4 wks, and 8 wks, total body, thigh LM and muscle characteristics were measured via dual‐energy x‐ray absorptiometry and B‐mode ultrasound, respectively. In a subsample, changes in WBPT was measured using [N^15^]alanine. Differences between groups were assessed using linear mixed models adjusted for baseline values, followed by 95% confidence intervals on adjusted mean change scores (Δ). HIIT and HIIT + EAA improved thigh LM (Δ: +0.17 ± 0.05 kg [0.08, 0.27]; +0.22 ± 0.05 kg [0.12,0.31]) and vastus lateralis cross‐sectional area (Δ: +2.73 ± 0.52 cm^2^ [1.69,3.77]; +2.64 ± 0.53 cm^2^ [1.58,3.70]), volume (Δ: +54.50 ± 11.69 cm^3^ [31.07, 77.92]; +62.39 ± 12.05 cm^3^ [38.26, 86.52]), and quality (Δ: −5.46 ± 2.68a.u. [−10.84, −0.09]; −7.97 ± 2.76a.u.[−13.49, −2.45]). Protein synthesis, breakdown, and flux were greater with HIIT + EAA and EAA compared to HIIT (*p* < .05). Sex differences were minimal. Compared to women, men tended to respond more to HIIT, with or without EAA. For women, responses were greater with HIIT + EAA than HIIT. In overweight and obese adults, 8 weeks of HIIT, with or without EAA, improved thigh LM size and quality; EAA may enhance muscular adaptation via increases in protein turnover, supporting greater improvements in muscular size and quality.

## Graphical abstract

In overweight and obese adults, eight weeks of high‐intensity interval training, with or without essential amino acid supplementation (EAA), improved thigh lean mass size and quality; EAA may enhance muscular adaptation via increases in protein turnover, supporting greater improvements in muscular size and quality.

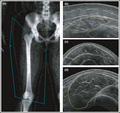

## 1 INTRODUCTION

The importance of lean body mass in the context of exercise performance, strength, and functionality is well recognized, but in the context of weight loss and metabolic health, the benefits of maintaining high‐quality lean mass (LM) are less commonly emphasized (Wolfe, 2006). Relative decreases in LM begin to occur at around the age of 30, with noticeable decreases occurring at around 45–50 years (Janssen et al., 2000). A loss of LM is associated with decreases in energy expenditure, reduced function and strength, and an increased risk of weight regain (Fothergill et al., 2016; Wolfe, 2006). The rate of age‐related loss in LM has been shown to be greater in the lower body/legs (Goodpaster et al., 2006; Janssen et al., 2000). Greater leg LM has previously been shown to be associated with a higher resting metabolic rate in both men and women (Hirsch et al., 2017). In addition to LM quantity, muscle quality also declines with age and obesity. Poor muscle quality, as a result of an increase in noncontractile tissue (i.e., intramuscular fat and connective tissue), is associated with impaired insulin sensitivity, muscle strength, and functionality (Corcoran et al., 2007; Mota et al., 2017; Rech et al., 2014; Wolfe, 2006). Effective strategies that support and promote the maintenance of skeletal muscle size and quality may have an important impact on metabolic disease (Wolfe, 2006).

High‐intensity interval training (HIIT), defined as bouts of vigorous exercise interspersed with periods of low‐intensity exercise or rest, is an efficient form of exercise known for its rapid improvements in cardiorespiratory fitness (Gibala et al., 2012; Metcalfe et al., 2012). Due to the significant effects of interval training on mitochondrial and cardiorespiratory adaptation, research has focused primarily on weight and fat loss with HIIT. However, recent studies have also reported increases in LM and muscle size (Blue et al., 2017; Gillen et al., 2013; Heydari et al., 2012; Macpherson et al., 2011; Moghaddam et al., 2020; Smith‐Ryan et al., 2015, 2016). After 3 weeks of HIIT, increases in 1.9 kg and 2.2 kg of total body LM were reported in overweight and obese men and women, respectively (Smith‐Ryan et al., 2015, 2016). Although these increases in total body LM were nonsignificant, follow‐up analysis showed a significant increase in muscle cross‐sectional area (mCSA) of the vastus lateralis (+3.2 cm^2^) (Blue et al., 2017). Other studies have reported significant increases in leg LM following 6 (+0.4 kg) and 12 weeks (+0.4 kg) of HIIT in both overweight men and women, despite nonsignificant or low‐magnitude changes in total body LM (+0.6–1.2 kg) (Gillen et al., 2013; Heydari et al., 2012). Assessment of regional changes in muscle characteristics may be a more descriptive and sensitive measure of muscular change associated with HIIT.

Ultrasonography has grown in popularity as a noninvasive approach to evaluating muscle characteristics. Unlike most body composition devices that estimate total and regional LM, ultrasound allows for the assessment of individual muscles. In addition to quantification of mCSA, ultrasonography can also be used to evaluate muscle quality, via grayscale analysis of echo intensity which estimates the amount of contractile and noncontractile tissue within a muscle, as well as architectural features, such as fascicle length and pennation angle, that influence muscular strength and force production (Ahtiainen et al., 2010; Narici et al., 2016; Young et al., 2015). These muscle characteristics provide insight into muscular health and functionality, but have been minimally evaluated in response to HIIT (Blackwell et al., 2020; Blue et al., 2017; Moghaddam et al., 2020).

Protein, specifically essential amino acids (EAA), is essential for muscular adaptation in response to exercise (Hulmi et al., 2010; Wolfe, 2001). When consumed prior to or following resistance exercise, EAAs augment muscle protein synthesis, resulting in an increase in muscle size (Areta et al., 2013; Borsheim et al., 2002; Hulmi et al., 2010; Rasmussen et al., 2000; Tipton et al., 2001a, 2001b; Wolfe, 2001). Few studies have evaluated the effects of HIIT in combination with a nutritional intervention (Gillen et al., 2013); to our knowledge, none have evaluated the effect of HIIT + EAA on LM and muscle characteristics. Improvements in LM with a nutrition and exercise intervention that requires minimal time and lifestyle changes, such as this, could have significant implications for improving and maintaining muscular health in a variety of populations. Therefore, the purpose of this study was to compare the independent and combined effects of HIIT and EAA supplementation on total body and thigh LM, muscle characteristics, and 24‐hr whole‐body protein turnover (WBPT) in overweight and obese men and women. Muscle characteristics include CSA of the superficial quadricep muscles (rectus femoris, vastus medialis, vastus lateralis), in addition to vastus lateralis quality, volume, and architectural characteristics. An exploratory aim was to evaluate the potential modulatory effects of sex. It was hypothesized that HIIT would increase thigh LM size and quality, as determined by changes in mCSA, muscle volume (MV), and echo intensity of the superficial quadriceps muscles; no changes in architectural characteristics of the vastus lateralis were predicted. It was also hypothesized that the addition of EAA would increase whole‐body protein balance, supporting greater improvements in LM, mCSA, and muscle quality, compared to HIIT alone. Finally, it was hypothesized that improvements would occur in both men and women, with greater changes occurring in men (Scalzo et al., 2014; Smith‐Ryan et al., 2015).

## 2 METHODS

### 2.1 Participants

Of the original 651 individuals who expressed initial interest in participation, 89 met initial inclusion criteria and completed an in‐person enrollment visit. Of these 89, five individuals did not meet inclusion criteria and were excluded at the enrollment visit, 14 individuals did not return for baseline testing following the enrollment visit, and four individuals completed baseline testing, but withdrew before completing mid‐ or post‐testing, resulting in 66 overweight and obese men (*N* = 33) and women (*N* = 33) between the ages of 25–50 years who participated in this study (Race: 69% White, 13% Black, 4% Hispanic, 3% Asian, 11% two or more races; Age: 36.7 ± 6.0 years; Height: 171.4 ± 9.8 cm; Weight: 94.5 ± 14.7 kg; %BF: 38.8 ± 7.2%; Table 1). For men, overweight/obese was defined as a body mass index (BMI) of 28–40 kg/m^2^ and/or body fat percentage (%BF) ≥ 25% and for women as a BMI of 25–40 kg·m^‐2^ and/or %BF ≥ 30% (Shah & Braverman, 2012). %BF was confirmed using bioelectrical impedance analysis (InBody770, BioSpace, Seoul, South Korea). Women were eumenorrheic, reporting consistent menstruation for three months prior to enrollment and confirmed not‐pregnant by a urine pregnancy test. Participants were otherwise healthy (no cardiovascular, metabolic, musculoskeletal, or surgical events within six months of enrollment), nonsmokers, participating in less than 150 min per week of moderate exercise, less than 2 days per week of resistance training, and had not participated in HIIT within 12 weeks prior to enrollment. Participants were also instructed to maintain habitual lifestyle and physical activity for the duration of the study. Detailed descriptions of CONSORT, inclusion/exclusion criteria, and participant demographics have been reported (Hirsch et al., 2020).

**Table 1:** Subject characteristics (Mean ± *SD*)

Total Group	HIIT (*N* = 19)	EAA (*N* = 20)	HIIT + EAA (*N* = 19)	CON (*N* = 8)
Age (yrs)	36.74 ± 5.61	37.20 ± 5.52	36.21 ± 6.65	36.88 ± 7.45
Height (cm)	173.76 ± 10.12	169.26 ± 8.91	170.64 ± 10.52	173.28 ± 9.51
Weight (kg)	96.57 ± 17.23	95.91 ± 13.19	91.78 ± 13.54	92.20 ± 15.52
BMI (kg/m2)	31.73 ± 4.72	33.52 ± 4.42	31.41 ± 3.36	30.55 ± 3.91
%BF	38.78 ± 5.92	40.31 ± 8.36	37.77 ± 6.91	37.66 ± 8.19
FM (kg)	37.31 ± 8.95	38.67 ± 10.23	34.44 ± 8.44	34.47 ± 9.56
LM (kg)	55.72 ± 10.93	53.74 ± 9.31	53.99 ± 10.36	54.31 ± 12.36
Thigh LM (kg)	7.25 ± 1.65	7.08 ± 1.37	7.09 ± 1.50	7.25 ± 2.03
RF mCSA (cm^2^)	11.08 ± 3.67	10.60 ± 2.24	10.59 ± 2.21	10.55 ± 2.03
VM mCSA (cm^2^)	21.24 ± 5.88	21.20 ± 6.16	19.10 ± 4.57	18.56 ± 7.58
VL mCSA (cm^2^)	24.69 ± 6.20	24.78 ± 6.24	25.12 ± 5.79	25.47 ± 7.31
EI (a.u.)	132.96 ± 35.81	144.78 ± 45.83	137.03 ± 39.93	135.72 ± 43.03
MV (cm^3^)	567.32 ± 163.92	558.95 ± 145.14	544.74 ± 158.76	603.63 ± 211.28

MALES	HIIT (*N* = 9)	EAA (*N* = 10)	HIIT + EAA (*N* = 10)	CON (*N* = 4)
Age (yrs)	36.67 ± 5.96	35.60 ± 4.95	37.30 ± 7.65	39.00 ± 10.80
Height (cm)	181.57 ± 6.09	175.51 ± 6.53	178.01 ± 8.11	180.63 ± 3.77
Weight (kg)	107.24 ± 13.02	96.66 ± 16.33	98.94 ± 10.07	101.48 ± 13.45
BMI (kg/m2)	32.69 ± 5.44	31.22 ± 4.29	31.14 ± 2.20	31.13 ± 5.00
%BF	35.67 ± 4.81	33.15 ± 4.95	32.96 ± 3.10	32.08 ± 7.14
FM (kg)	38.56 ± 9.43	32.41 ± 9.58	32.58 ± 5.44	32.96 ± 11.30
LM (kg)	64.78 ± 3.91	60.64 ± 7.67	62.63 ± 5.47	64.96 ± 6.70
Thigh LM (kg)	8.64 ± 0.69	7.99 ± 1.29	8.30 ± 0.90	8.94 ± 1.22
RF mCSA (cm^2^)	12.79 ± 2.70	11.14 ± 2.91	11.85 ± 2.10	10.53 ± 2.73
VM mCSA (cm^2^)	25.33 ± 4.31	25.11 ± 5.78	22.03 ± 4.25	24.20 ± 6.29
VL mCSA (cm^2^)	28.39 ± 5.37	27.97 ± 5.64	27.97 ± 6.14	30.27 ± 7.30
EI (a.u.)	106.85 ± 20.34	107.96 ± 22.23	109.05 ± 15.81	101.95 ± 8.92
MV (cm^3^)	704.39 ± 81.13	650.31 ± 139.38	656.33 ± 130.47	769.43 ± 169.79

FEMALES	HIIT (*N* = 10)	EAA (*N* = 10)	HIIT + EAA (*N* = 9)	CON (*N* = 4)
Age (yrs)	36.80 ± 5.59	38.80 ± 5.85	35.00 ± 5.52	34.75 ± 0.96
Height (cm)	166.74 ± 7.49	163.01 ± 6.18	162.46 ± 5.64	165.93 ± 7.25
Weight (kg)	86.97 ± 15.06	95.15 ± 9.95	83.82 ± 12.76	82.93 ± 12.33
BMI (kg/m^2^)	30.86 ± 4.07	35.82 ± 3.32	31.70 ± 4.45	29.98 ± 3.12
%BF	41.59 ± 5.57	47.47 ± 3.03	43.11 ± 5.97	43.25 ± 4.75
FM (kg)	36.18 ± 8.85	44.94 ± 6.49	36.51 ± 10.85	35.97 ± 8.91
LM (kg)	47.58 ± 8.36	46.83 ± 4.26	44.39 ± 3.29	43.67 ± 3.09
Thigh LM (kg)	6.00 ± 1.16	6.16 ± 0.68	5.74 ± 0.53	5.56 ± 0.73
RF mCSA (cm^2^)	9.54 ± 3.85	10.05 ± 1.21	9.20 ± 1.38	10.56 ± 1.48
VM mCSA (cm^2^)	17.56 ± 4.58	17.29 ± 3.55	15.84 ± 2.03	12.91 ± 3.05
VL mCSA (cm^2^)	21.37 ± 5.04	21.59 ± 5.26	21.96 ± 3.42	20.67 ± 3.17
EI (a.u.)	156.47 ± 30.03	181.60 ± 30.46	168.11 ± 35.23	169.49 ± 34.62
MV (cm^3^)	443.95 ± 110.39	467.60 ± 80.64	420.74 ± 68.55	437.82 ± 44.94

Abbreviations: BMI, body mass index; %BF, percent body fat; FM, fat mass; LM, lean mass; mCSA, muscle cross‐sectional area; RF, rectus femoris; VM, vastus medialis; VL, vastus lateralis; EI, echo intensity; MV, muscle volume.

### 2.2 Experimental Design

Using 2:2:2:1 block randomization, with equal allocation of men and women, individuals were randomly assigned to 8 weeks of: (a) HIIT, two days/week of cycle ergometry; (b) EAA supplementation, consuming 3.6 grams EAA twice daily; (c) HIIT + EAA; or (d) control (CON), maintaining normal diet and exercise habits. Measurements of body composition and muscle size, quality, and architectural characteristics were measured at baseline, 4 weeks, and 8 weeks. Whole‐body protein turnover was measured in a subsample of individuals from the HIIT (*N* = 8), EAA (*N* = 7), and HIIT + EAA (*N* = 7) groups at baseline and 8 weeks. All participants provided written informed consent, completed a health history questionnaire to confirm inclusion/exclusion criteria, and underwent a 12‐lead electrocardiogram prior to baseline testing. Participants were asked to abstain from food and caloric beverages (12hrs), caffeine (12hrs), alcohol (24hrs), and physical activity (24hrs) prior to testing sessions. Participants were also asked to remove all metal, plastics, and heavy clothing upon arrival, to avoid interference with body composition measures. All procedures were approved by the University Biomedical Institutional Review Board.

### 2.3 Procedures

#### 2.3.1 High‐Intensity Interval Training

**Figure 1:**
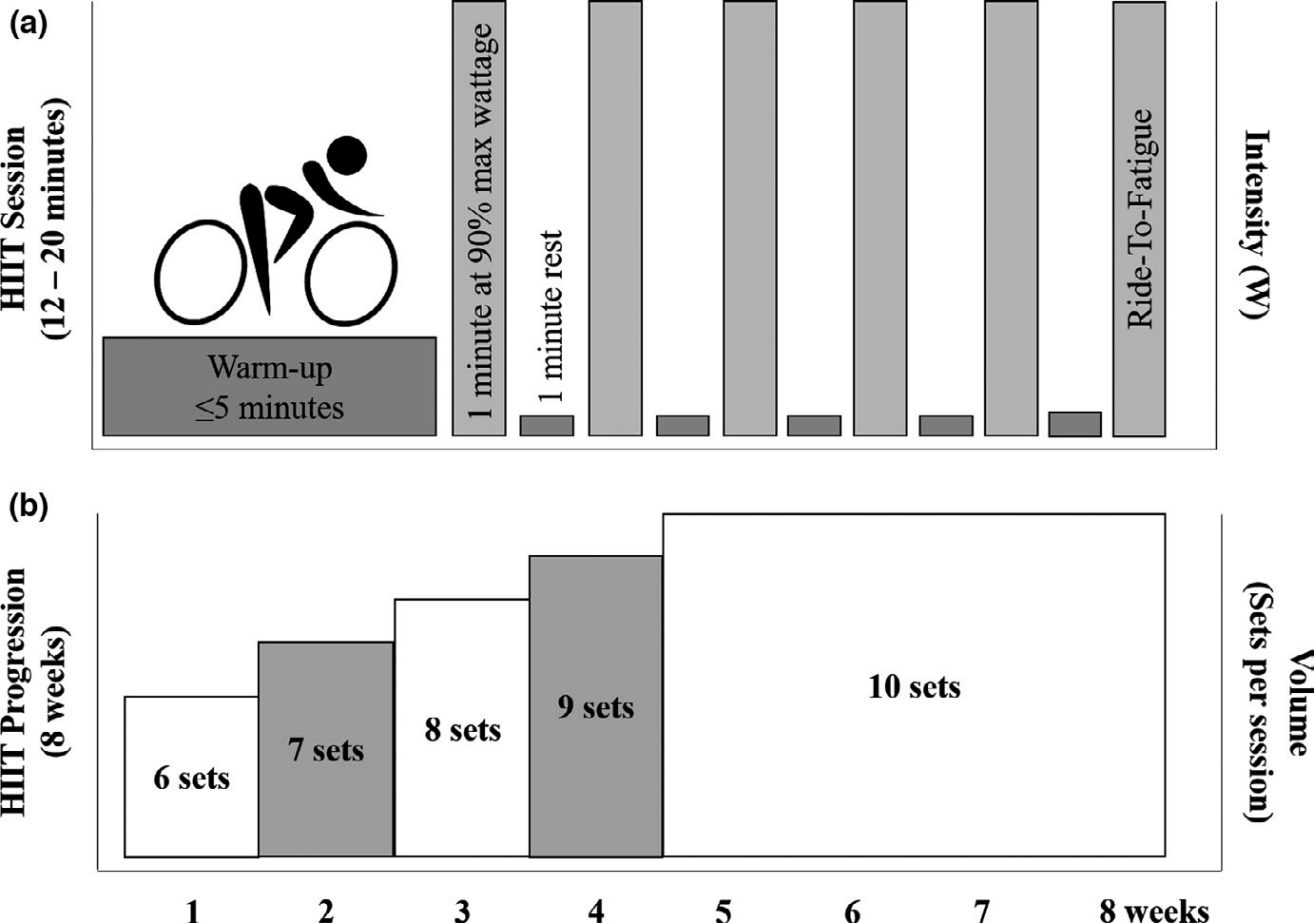
(a) Timeline of a single HIIT session. Each session started with a self‐selected warm‐up, followed by alternating sets of 1 min of hard pedaling (90% of max watts) and 1‐min rest. During the final repetition, individuals were asked to pedal as long as possible. If ride duration was > 75 s total, resistance was increased by 7% at the next session; if the ride duration was ≤ 75 s, resistance was maintained for the next session. (b) Progression of HIIT over the course of the 8‐week intervention. The intervention started with six sets of intervals. One set was added each week until reaching 10 sets at week five; 10 sets were maintained for the remainder of the 8 weeks

All training occurred on a cycle ergometer, two days per week for 8 weeks, with one‐on‐one supervision. Each session consisted of a self‐selected warm‐up (≤5 min), followed by alternating sets of one minute at 90% max wattage and one‐minute recovery at complete rest. Training started with six sets of intervals and progressed by one set each week until reaching 10 sets at week five; 10 sets were maintained for the remainder of the 8 weeks (Figure 1). To maintain an appropriate individualized high‐intensity workload, individuals were asked to ride to fatigue on the last set of each session. If the individual was able to ride for > 75 s, resistance was increased by 7% at the next session; if the individual rode for  ≤ 75 s, resistance was maintained for the next session (based on unpublished pilot data) (Figure 1). Training sessions were separated by at least 24 hr, with preferential scheduling on nonconsecutive days. Starting intensity was individualized for each participant based on maximum wattage reached during baseline cardiovascular fitness (VO_2_peak) testing, as previously described (Hirsch et al., 2020). Exercise volume was calculated for each HIIT session (volume = workload_watts_ × [total time exercising]) and summed together to determine exercise volume from 0–4, 4–8, and 0–8 weeks. Exercise volume results have been reported (Hirsch et al., 2020). Although not significantly different, HIIT + EAA had a higher overall exercise volume than HIIT (difference at 8 weeks = 2081.66 ± 2912.13 watt·min; *p* = .479). Based on the number of sessions completed, adherence in both exercise groups (HIIT, HIIT + EAA) for the entire 8 weeks was 96%; for weeks 0–4 and 4–8, average adherence was 98% and 95%, respectively.

#### 2.3.2 Essential Amino Acid Supplementation

The EAA supplement contained 3.6 g of a patented‐ratio blend of L‐leucine, L‐lysine HCl, L‐valine, L‐isoleucine, L‐arginine, L‐threonine, L‐phenylalanine, L‐methionine, L‐histidine, and L‐tryptophan (REAAL, Twinlab Corporation, Hauppauge, NY, USA). Participants were instructed to consume the EAA powder mixed with water (8–12 oz), two times per day between meals; one serving between the hours of 9:00a.m.–12:00p.m. and the second serving between the hours of 3:00p.m.–11:00p.m., with at least 3 hr between doses. On training days, participants assigned to the HIIT + EAA group consumed one serving 30 min prior to and following exercise. Participants were given a log to record supplement consumption at home. Supplement containers were also collected and weighed at 4 weeks and 8 weeks to track compliance. Based on percentage of prescribed supplement that was consumed, average adherence in both supplement groups (EAA, HIIT + EAA) for the entire 8 weeks was 89%; for weeks 0–4 and 4–8, average adherence was 91% and 85%, respectively.

#### 2.3.3 Total and Regional Body Composition

**Figure 2:**
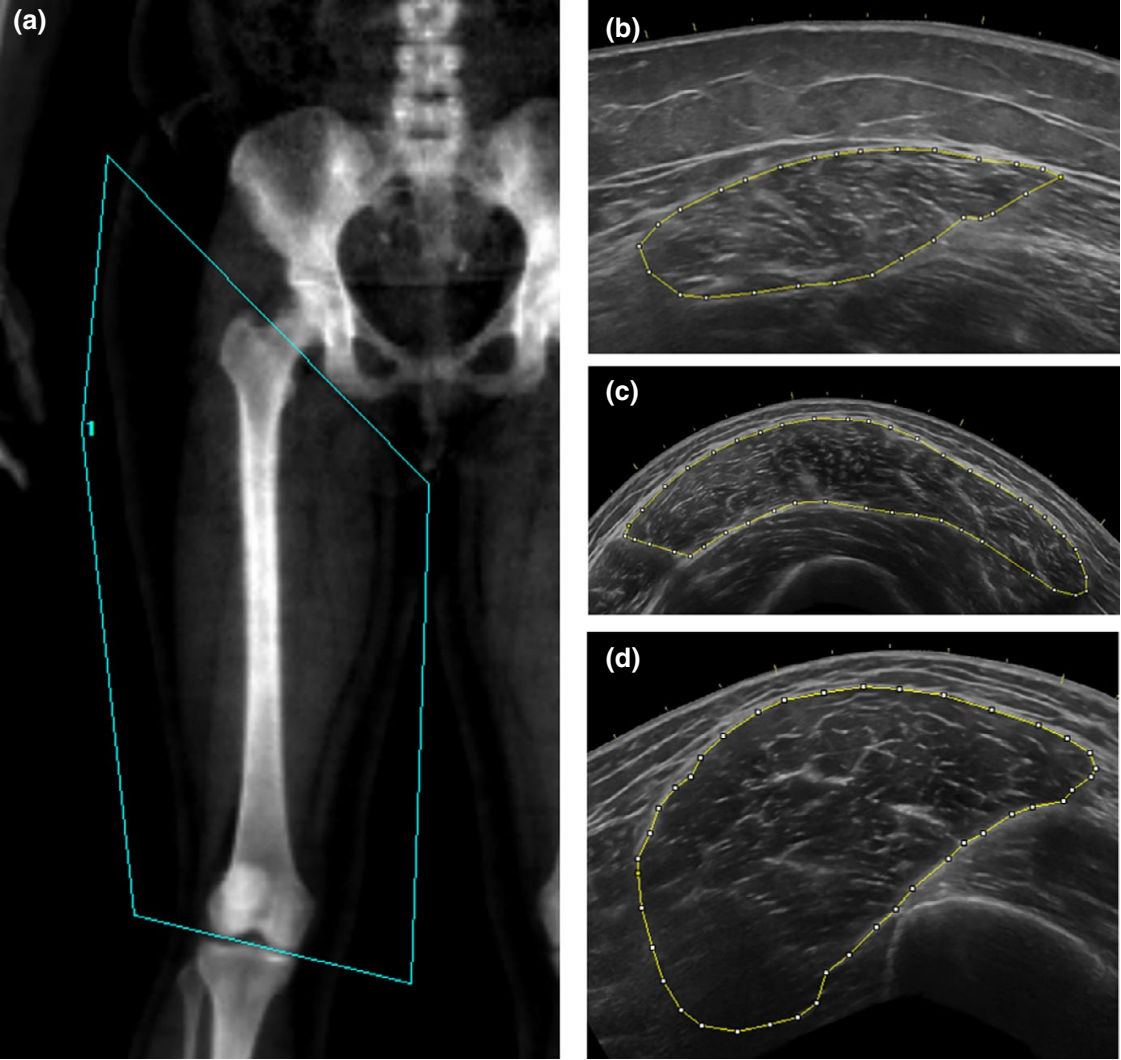
(a) Thigh lean mass region‐of‐interest. Ultrasound cross‐sectional area of the (b) rectus femoris, (c) vastus lateralis, and (d) vastus medialis

Body composition, specifically total body LM and thighLM, were measured from a total body dual‐energy x‐ray absorptiometry scan (DXA; GE Lunar iDXA, GE Medical Systems Ultrasound & Primary Care Diagnostics, Madison, WI, USA). For sub‐analysis of thighLM, a region‐of‐interest (ROI) was manually drawn around the right thigh, such that, (a) the thigh was separated from trunk by a line bisecting the femoral head and touching the ischial tuberosity, as would be drawn to form the pelvic triangle; and (b) the thigh was separated from the lower shank by a line drawn bisecting the intercondylar space between the femur and the tibia (Figure 2a). All scans were performed and analyzed by a trained technician, following manufacturer guidelines and using manufacturer software (enCORE Software Version 16). Test–retest reliability for DXA measurements is as follows: LM (ICC = 0.998, *SEM* = 0.806 kg); thighLM (ICC_2,1_ = 0.999, *SEM* = 0.196 kg).

#### 2.3.4 Muscle Characteristics

Muscle CSA of the vastus lateralis (VL), rectus femoris (RF), and vastus medialis (VM) was determined from panoramic ultrasound (US) scans (GE LOGIQ‐e, Software version R8.0.7, GE Healthcare, Wisconsin, USA) (Figure 2) using a linear array US transducer probe (GE: 12L‐RS) and standardized frequency (10 Hz) and gain (50) settings ( Hirsch et al., 2016; Melvin et al., 2014). Measurements were made by applying the device probe directly against the skin at the peak anatomical CSA of each muscle, defined as 30%, 50%, and 60% of femur length for the VM, VL, and RF, respectively (Hogrel et al., 2015; Morse et al., 2007).

Muscle volume (MV) was evaluated from cross‐sectional scans of the VL taken at 25%, 50%, and 75% of muscle length using the cylinder method, as previously described (Hogrel et al., 2015; Morse et al., 2007). Pennation angle (PA) and fascicle length (FL) of the VL were also evaluated from panoramic scans along the fascicular plane at 50% of femur length (Gerstner et al., 2017). The scans were performed by the same technician while the subject laid supine with the right leg extended and relaxed on the examination table for approximately 5 min.

All images were exported and analyzed using Image‐J software (National Institutes of Health, USA, version 1.51). Muscle CSA was determined by tracing the outline of the muscle along the inside fascial border (Hirsch et al., 2016; Melvin et al., 2014). Echo intensity (EI), an ultrasound‐derived measure of muscle quality, was determined using grayscale analysis, with a correction for subcutaneous fat thickness [EI = EI_raw_ + (SAT × 40.5278)] from the cross‐sectional image of the VL taken at 50% of femur length (Hirsch et al., 2016; Melvin et al., 2014; Young et al., 2015). Fascicle length was determined as the length of one fascicle between the superficial and deep aponeuroses, measured near the center of the image (Gerstner et al., 2017); PA was determined by measuring the angle between the deep aponeurosis and from the same fascicle used to determine FL (Gerstner et al., 2017). The same technician performed all analyses for each outcome. Each image was individually calibrated by measuring the number of pixels in a known distance (image depth). Two images from each location were analyzed and an average of the two measures was reported for all outcomes (CSA, EI, FL, PA). Test–retest reliability for mCSA and EI from our laboratory is as follows: mCSA (ICC = 0.99, *SEM* of 0.744 cm^2^); EI (ICC = 0.99, *SEM* = 1.5 a.u).

#### 2.3.5 Whole‐Body Protein Turnover

Whole‐body protein turnover (g N/24hr) was determined by [^15^N]alanine isotope tracer (98% enriched, Cambridge Isotope Lab, Andover, MA) (Ferrando et al., 1996) in which participants ingested a 2.00 gram dose of [^15^N]alanine mixed with water. For the 24hrs following ingestion, participants were asked to collect urine from all voids and keep a diet record of all food and drink consumed. Diet records were analyzed for protein intake (g) to account for dietary nitrogen intake. Zero and 24‐hr blood draws were collected to measure blood urea nitrogen. Isotopically labeled nitrogen from the blood and urine samples was used to determine nitrogen flux according to Fern et al. (1985). Whole‐body protein synthesis (PS) and breakdown (PB) were calculated from urine samples according to Stein et al. (1991) and used to determine net protein balance and flux. Samples were shipped and analyzed at the Center for Translational Research in Aging and Longevity, University of Arkansas Medical Sciences, Little Rock, AR.

#### 2.3.6 Dietary Intake

Three‐day dietary logs were collected at baseline, 4 weeks, and 8 weeks to account for the influence of normal dietary intake. Subjects were instructed to record all food and drink consumed on two, nonconsecutive weekdays and one weekend day. Detailed verbal and printed instructions were provided, instructing on how to complete the diet logs and estimate portion sizes. Diet logs were analyzed for average calories (CAL; kcal), carbohydrate (CHO; g), fat (FAT; g), protein (PRO; g) and relative protein (g/kg body mass) intake using nutrition analysis software (The Food Processor, version 10.12.0, Esha Research, Salem, OR, USA). Full dietary intake data have previously been reported (Hirsch et al., 2020). There were no differences in dietary intake for the full sample or in the subsample of individuals who completed the measure of WBPT (*p* > .05).

### 2.4 Statistical Analysis

A modified intent‐to‐treat analysis was conducted, including only participants who completed mid‐ (*N* = 66) and/or post‐testing (*N* = 62). Group‐by‐time interaction effects on total body LM and thigh LM, were evaluated using separate 4 × 2 [group (EAA versus. HIIT versus. HIIT + EAA versus. CON) × time (4wk versus. 8 wk)] linear mixed models, covaried for baseline values. Secondary outcomes, (mCSA, EI, MV, FL, PA) were also evaluated using 4 × 2 linear mixed models, covaried for baseline values; differences in whole‐body PS, PB, and NB at 8 weeks was evaluated using separate one‐way linear mixed model, covaried for baseline values. Significant main effects were followed by analysis of 95% confidence intervals (CI) on the mean change scores adjusted for baseline values to assess changes from 0–4, 4–8, and 0–8 weeks. The mean change score was considered statistically significant if the 95% CI interval did not include zero.

To explore the modulatory effects of sex, separate group‐by‐time‐by‐sex interaction effects on total body LM, thighLM, and muscle size, quality, and architecture characteristics were evaluated using separate 4 × 2 × 2 (group × time ×sex) linear mixed models, covaried for baseline values, followed by 95% CI to evaluate change, using the same procedures as described above. All statistical computations were performed using SPSS (Version 21, IBM, Armonk, NY, USA), using an α = 0.05 to determine statistical significance.

## 3 RESULTS

### 3.1 Total Body and Thigh Lean Mass

**Figure 3:**
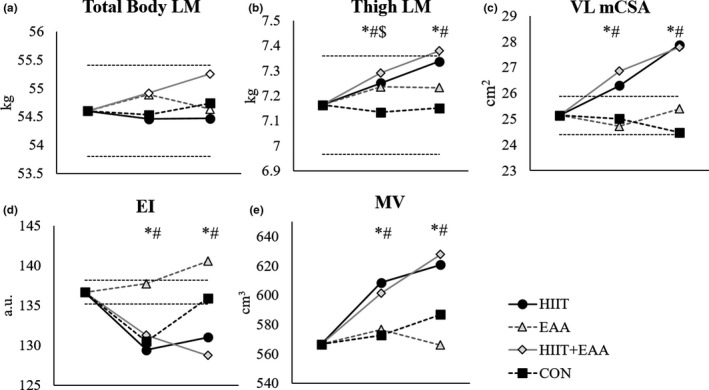
Adjusted means for (a) total body lean mass, (b) thigh lean mass, (c) vastus lateralis cross‐sectional area, (d) vastus lateralis echo intensity, and (e) vastus lateralis muscle volume at baseline, 4 weeks, and 8 weeks; dotted lines represent ± standard error of the measure. Significant change for *HIIT + EAA, #HIIT, and $EAA based on 95% CI on adjusted mean change

For total body LM, there was no significant interaction (*p* = .654) or main effects for group (*p* = .771) or time (*p* = .076) (Figure 3a). For thigh LM, there was no interaction (*p* = .636) or main effect for time (*p* = .176), but there was a main effect for group (*p* = .003). Analysis of 95% CI showed increases in thigh LM from weeks 0 to 4 for HIIT, EAA, and HIIT + EAA (Table 2); there were further increases for HIIT and HIIT + EAA from weeks 4 to 8, resulting in overall increases in thigh LM from weeks 0 to 8 (Figure 3b). There were no changes for CON. There was no significant difference in change between groups (*p* > .05).

**Table 2:** Mean change scores adjusted for baseline values with 95% confidence intervals (mean ± SE [95%CI])

	Weeks	HIIT	EAA	HIIT + EAA	CON
Thigh LM	0–4	0.09 ± 0.04 [0.01,0.16]*	0.07 ± 0.04 [0.001,0.15]*	0.13 ± 0.04 [0.06,0.20]*	−0.03 ± 0.06 [−0.14,0.09]
(kg)	4–8	0.09 ± 0.04 [0.01,0.16]*	−0.00 ± 0.04 [−0.08,0.08]	0.09 ± 0.04 [0.01,0.17]*	0.02 ± 0.06 [−0.10,0.14]
	0–8	0.17 ± 0.05 [0.08,0.27]*	0.07 ± 0.05 [−0.03,0.17]	0.22 ± 0.05 [0.12,0.31]*	−0.01 ± 0.07 [−0.16,0.13]
VL mCSA	0–4	1.16 ± 0.51 [0.13,2.18]*	−0.41 ± 0.50 [−1.41,0.59]	1.73 ± 0.51 [0.70,2.75]*	−0.12 ± 0.80 [−1.70,1.46]
(cm^2^)	4–8	1.59 ± 0.44 [0.72,2.47]*	0.45 ± 0.46 [−0.48,1.38]	0.74 ± 0.45 [−0.16,1.64]	−0.53 ± 0.67 [−1.88,0.82]
	0–8	2.73 ± 0.52 [1.69,3.77]*	0.25 ± 0.55 [−0.85,1.34]	2.64 ± 0.53 [1.58,3.70]*	−0.66 ± 0.80 [−2.26,0.93]
EI	0–4	−7.47 ± 2.51 [−12.49,−2.45]*	1.20 ± 2.45 [−3.70,6.10]	−5.51 ± 2.51 [−10.52,−0.50]*	−6.31 ± 3.86 [−14.03,1.41]
(a.u.)	4–8	1.74 ± 2.60 [−3.46,6.94]	2.31 ± 2.75 [−3.18,7.81]	−2.70 ± 2.67 [−8.04,2.65]	5.38 ± 4.00 [−2.63,13.39]
	0–8	−5.46 ± 2.68 [−10.84,−0.09]*	4.02 ± 2.84 [−1.66,9.70]	−7.97 ± 2.76 [−13.49,−2.45]*	−0.67 ± 4.13 [−8.95,7.61]
MV	0–4	41.90 ± 10.23 [21.44,62.36]*	9.65 ± 9.97 [−10.30,29.59]	33.73 ± 10.25 [13.24,54.23]*	7.55 ± 15.83 [−24.11,39.21]
(cm^3^)	4–8	12.49 ± 8.28 [−4.09,29.08]	−2.36 ± 8.76 [−19.90,15.19]	26.90 ± 8.53 [9.82,43.99]*	11.88 ± 12.80 [−13.75,37.51]
	0–8	54.50 ± 11.69 [31.07,77.92]*	−0.90 ± 12.38 [−25.68,23.88]	62.39 ± 12.05 [38.26,86.52]*	19.15 ± 18.07 [−17.04,55.35]

Abbreviations: EI, echo intensity; MV, muscle volume of the VL; Thigh LM, thigh lean mass; VL mCSA, vastus lateralis muscle cross‐sectional area.

* Significant change based on 95% CI.

### 3.2 Muscle Cross‐Sectional Area

There was no significant interaction effect for mCSA of the RF (*p* = .976), VM (*p* = .781), or VL (*p* = .463). There were no main effects for RF (*p* = .284–0.836); for the VM, there was a main effect for group (*p* = .044), but not time (*p* = .952) and for the VL there was a significant main effect for group (*p* < .001) and time (*p* = .031). For the VM, 95% CI showed no significant changes from weeks 0–4 or 4–8, but an increase in mCSA for CON from weeks 0 to 8 (∆: 1.59 ± 0.79 cm^2^; [0.01,3.18]). For the VL, 95% CI showed a significant increase in VL mCSA from weeks 0 to 4 for HIIT and HIIT + EAA, a significant increase from weeks 4 to 8 for HIIT, and from weeks 0 to 8 for HIIT and HIIT + EAA (Figure 3c; Table 1). No changes were observed for EAA or CON.

### 3.3 Muscle Quality

For EI of the VL, there was no significant interaction (*p* = .626) or main effect for time (*p* = .539), but there was a significant main effect for the group (*p* = .002). Analysis of 95% CI showed improvements in muscle quality for HIIT and HIIT + EAA from weeks 0–4 and 0–8 (Figure 3d; Table 2). There were no significant changes in EI from weeks 4 to 8; there were no significant changes in EI for EAA or CON.

### 3.4 Muscle Volume

For MV of the VL, there was no significant interaction (*p* = .421) or main effect for time (*p* = .238), but there was a main effect for group (*p* < .001). Analysis of 95% CI showed significant increases in MV from weeks 0 to 4 for HIIT and HIIT + EAA, further increases from weeks 4 to 8 for HIIT + EAA, resulting in significant increases in MV from weeks 0 to 8 for HIIT and HIIT + EAA (Figure 3e; Table 2). Changes between HIIT and HIIT + EAA were not significantly different (*p* = 1.000). There were no significant changes for EAA or CON.

### 3.5 Muscle architecture

There was no significant interaction (*p* = .682–0.804) or main effects (*p* = .142–0.834) for FL or PA (Table 3).

**Table 3:** Muscle architectural features (Mean ± *SD*)

	Week	HIIT	EAA	HIIT + EAA	CON
VL FL	0	7.07 ± 1.28	7.24 ± 1.11	7.19 ± 1.05	8.26 ± 0.51
(cm)	4	7.31 ± 1.21	7.42 ± 0.84	7.46 ± 1.10	8.10 ± 0.57
	8	7.35 ± 1.18	7.13 ± 0.75	7.57 ± 0.93	7.95 ± 0.91
VL PA	0	18.08 ± 2.42	17.91 ± 3.10	18.98 ± 3.26	15.68 ± 3.31
(°)	4	17.82 ± 3.09	17.04 ± 3.15	19.27 ± 3.16	15.59 ± 2.62
	8	17.67 ± 2.99	17.67 ± 2.10	19.15 ± 3.27	16.64 ± 3.59

Note

No significant differences between groups or time (*p* > .05). FL, fascicle length; PA, pennation angle.

### 3.6 Whole‐body protein turnover

In the subsample of individuals who completed the measure of whole‐body protein turnover (*n* = 22), net balance significantly decreased from weeks 0 to 8 for HIIT + EAA and EAA, after adjusting for baseline values (Table 4). However, both groups remained in protein balance, with no difference in net balance between groups at 8 weeks (*p* = .157). Whole‐body protein synthesis significantly decreased from weeks 0 to 8 for HIIT, resulting in lower protein synthesis for HIIT compared to HIIT + EAA and EAA at 8 weeks (*p* < .05) (Table 4). Protein breakdown did not significantly change from weeks 0 to 8 for any group, but breakdown tended to be greater with HIIT + EAA and EAA at 8 weeks compared to HIIT (significant group effect: *p* = .032). Similarly, flux did not significantly change from weeks 0 to 8 for any group, but flux tended to be greater with HIIT + EAA and EAA at 8 weeks compared to HIIT (significant group effect: *p* = .024).

**Table 4:** Change in whole‐body protein turnover from 0 to 8 weeks (mean ± SE [95% CI])

	HIIT	EAA	HIIT + EAA
PS* (g/kgBM/d)	−1.03 ± 0.48 [−2.04,−0.02]#	0.82 ± 0.52 [−0.27,1.92]	0.79 ± 0.52 [−0.31,1.89]
PB* (g/kgBM/d)	−0.87 ± 0.55 [−2.02,0.29]	1.21 ± 0.60 [−0.05,2.47]	1.06 ± 0.59 [−0.18,2.30]
NB (g/kgBM/d)	−0.02 ± 0.16 [−0.35,0.31]	−0.46 ± 0.16 [−0.81,−0.12]#	−0.36 ± 0.16 [−0.70,−0.02]#
Flux* (g/kgBM/d)	−1.04 ± 0.53 [−2.14,0.06]	1.01 ± 0.57 [−0.18,2.20]	0.90 ± 0.57 [−0.29,2.09]

Note

NB: net balance; values adjusted for baseline values.

* Significant main effect for group 8 weeks (*p* < .05).

^#^ Significant change based on adjusted mean change and 95% CI.

### 3.7 Sex differences

There was no group × time ×sex interaction for any outcome (*p* > .05), but there was a significant sex × group interaction effect for thigh LM (*p* = .003), EI (*p* = .021), MV (*p* = .008) and PA (*p* = .009); there was also a main effect for group (*p* < .001) and sex (*p* = .021) for VL mCSA.

**Figure 4:**
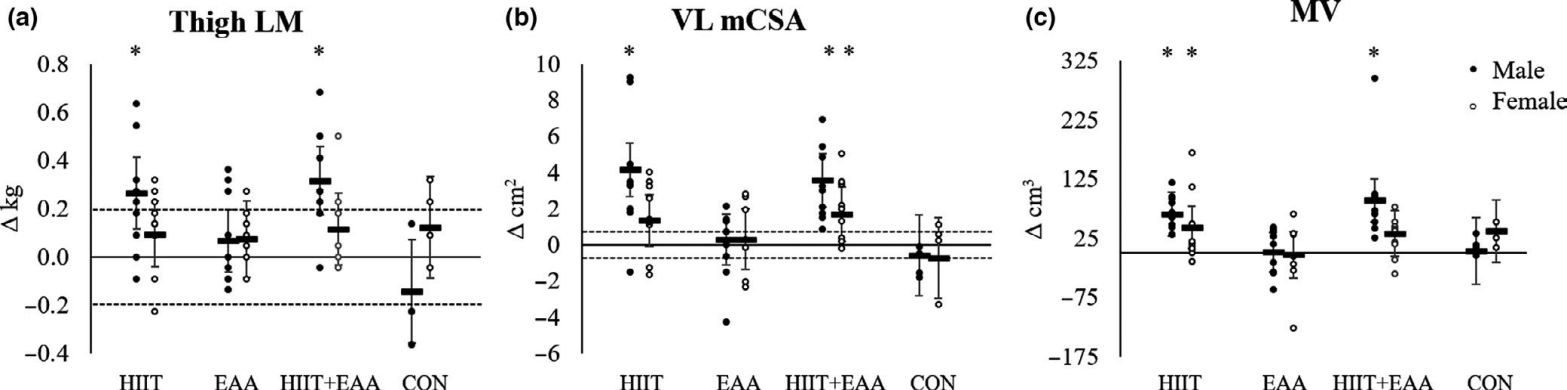
Mean and individual changes in thigh lean mass (a), vastus lateralis cross‐sectional area (b), and vastus lateralis muscle volume (c) for males and females from 0 to 8 weeks with 95% CI. Mean change scores are adjusted for baseline values. Dotted lines represent ± standard error of the measure. *significant change based on 95% CI

For thigh LM, analysis of 95% CI showed an increase for men with HIIT and HIIT + EAA from 4–8 weeks and 0–8 (Figure 4a); in women, thigh LM increased with HIIT + EAA from weeks 0 to 4 only. For muscle quality of the VL, men improved with HIIT + EAA from weeks 0 to 4 only, whereas in women, muscle quality improved with HIIT from weeks 0 to 4 and declined with EAA from weeks 0 to 8. For MV, there was a significant increase for men from weeks 0 to 4 for HIIT and HIIT + EAA, from weeks 4 to 8 with HIIT + EAA, resulting in significant increases from weeks 0 to 8 for HIIT and HIIT + EAA (Figure 4c); for women, MV increased from weeks 0 to 4 and 0–8 with HIIT only (Figure 4c). For PA, there was an increase in men from weeks 4–8 in CON (Δ: 2.10 ± 0.95°; [0.18,4.01]), whereas in women PA decreased from weeks 0 to 8 with HIIT (Δ: −1.63 ± 0.61°; [−2.85,‐0.40]). For mCSA of the VL, men increased with HIIT and HIIT + EAA from weeks 0–4, 4–8, and 0–8 (Figure 4b); for women, mCSA of the VL increased from weeks 0 to 4 with HIIT and HIIT + EAA, and from weeks 0 to 8 with HIIT + EAA (Figure 4b).

## 4 DISCUSSION

Previous studies have suggested that HIIT may promote increases in lean body mass and muscle size ( Blue et al., 2017; Gillen et al., 2013; Heydari et al., 2012; Macpherson et al., 2011; Moghaddam et al., 2020; Smith‐Ryan et al., 2015, 2016). To date, these reports have been inconsistent, exploratory in nature, and have not included a nutritional arm. Results of this study show that 8 weeks of HIIT improved muscle size and quality, as indicated by increases in thighLM, mCSA, MV, and decreased EI, respectively. Results also suggest that, with time, EAA supplementation may enhance muscular adaptation with HIIT, via an increase in protein turnover. Muscle responses did not significantly vary by sex. However, men tended to have greater muscular adaptations in response to HIIT and HIIT + EAA compared to women. For women, adaptations appeared more favorable with HIIT + EAA compared to HIIT.

Previous studies exploring the effect of HIIT on LM have predominately focused on changes in total body composition, the results of which have been inconsistent ( Wewege et al., 2017). Inconsistencies are likely related to exercise modality; HIIT training in the research setting is predominately conducted on a cycle ergometer, which almost exclusively targets the legs. This study uniquely examined changes in thigh LM, estimating change in the muscles specifically being targeted by HIIT. Despite minimal changes in total body LM, HIIT, and HIIT + EAA resulted in significant increases in thigh LM (HIIT: +0.17 kg; HIIT + EAA: +0.21 kg), specifically increasing mCSA (HIIT: +2.73 cm^2^; HIIT + EAA: +2.64 cm^2^) and MV (HIIT: +54.50 cm^3^; HIIT + EAA: +62.39 cm^3^) of the VL. Of the few studies that have evaluated regional changes in LM, significant increases in leg LM have been previously reported in overweight women (+0.4 kg) and men (+0.4 kg) after 6 and 12 weeks, respectively (Gillen et al., 2013; Heydari et al., 2012). After 3 weeks of HIIT, Blue et al. (2017) reported a nonsignificant 0.18 kg increase in leg LM and a significant 3.17 cm^2^ increase in mCSA of the VL in overweight and obese men and women, whereas Moghaddam et al. (2020) showed significant increases in mCSA of the VL (+1.2 cm^2^) and RF (+0.4 cm^2^) following 4 weeks of whole‐body HIIT in recreationally active young adults. Comparable increases were observed in this study, but occurred more gradually (over 8 weeks versus 3–4 weeks), likely due to the lower training frequency (2 days per week versus 3 days per week). More importantly, results are considered clinically significant and surpass measurement error (>0.74 cm^2^). Although less than what may be achieved with resistance training, the increases in thigh LM observed in this study would effectively offset annual age‐related declines in LM, which is estimated to be around 1.9 kg and 1.1 kg per decade for men and women, respectively, with a greater percentage of loss occurring in the legs (Janssen et al., 2000). Combined with improved muscle quality that was observed with HIIT and HIIT + EAA, suggesting a potential decrease in noncontractile tissues like intramuscular fat, improved muscle size and quality with HIIT and HIIT + EAA could have a significant long‐term impact on maintaining health, functionality, and quality of life.

Although changes in muscle size were not significantly different between HIIT and HIIT + EAA in this study, results do suggest that EAA may support greater increases in LM. On average, increases in total body LM, thigh LM, and MV were greater for HIIT + EAA (Figure 3) and it is possible that these differences would become more pronounced with time. A previous study in older adults showed gradual increases in LM with twice daily EAA supplementation over time, similar to that of this study, but changes did not reach significance until after 12 weeks (Borsheim et al., 2008). Although that study supplemented with a 11g dose of EAA, over three times greater than this study, there was no exercise intervention, which has stronger stimulatory effects on protein synthesis. Furthermore, the 3.6 g dose in this study has been shown to stimulate protein synthesis postexercise (Miller et al., 2003) and is a more feasible/realistic dosing strategy. Analysis of whole‐body protein turnover in this study also showed that protein synthesis decreased with HIIT, whereas protein synthesis was maintained with EAA. This resulted in greater protein synthesis for HIIT + EAA and EAA compared to HIIT at 8 weeks. Protein breakdown was also greater for HIIT + EAA and EAA at 8 weeks resulting in greater protein flux compared to HIIT only. This likely indicates greater protein turnover that is known to occur with the increased availability of amino acids (Wolfe, 2001, 2006), which would be supportive of LM remodeling. Finally, it is important to note that these results were achieved despite suboptimal daily dietary protein intake (0.9–1.0 g/kgBM d^−1^) for building muscle mass (1.4–2.0 g/kg d^−1^) (Jager et al., 2017). Therefore, it is likely that the minimal dose of EAA was enough time to support increases in LM, but 8 weeks was not enough to elucidate a clear effect.

Changes in muscle size can be accompanied by changes in muscle architecture which are associated with muscle strength and force production (Mangine et al., 2018), but to date, muscle architectural changes with HIIT training have been minimally evaluated. The results of this study showed minimal changes in muscle architecture. In contrast, an increase in PA (+2.5°), but not FL (−0.08 mm), accompanied by increases in muscle thickness, has previously been reported following 4 weeks of HIIT (3–4 d/wk) in cancer patients (Blackwell et al., 2020). Changes in muscle architecture are commonly seen following resistance training, which incorporates both concentric and eccentric muscle actions (Franchi et al., 2014). The lack of changes in architecture in this study potentially could be due to cycling being a primarily concentric exercise, which have been shown to be less of stimulus for morphological adaptation compared to eccentric exercise (Franchi et al., 2014). The lack of architectural changes also suggests that muscle hypertrophy in this study may be due to the expansion of nonforce‐generating components of muscle, as opposed to myofibrillar changes. Muscle hypertrophy following high‐volume resistance training has also been shown to be largely attributed to sarcoplasmic hypertrophy, as opposed to architectural changes (Haun et al., 2019). Sarcoplasmic expansion was shown to be associated with increased proteins involved with glycolysis and ATP generation, which would have beneficial effects for HIIT performance (Haun et al., 2019). It is currently unclear how sarcoplasmic expansion may influence strength/power and metabolic outcomes, but further research into the mode of hypertrophy and concurrent influences on strength, functionality, and metabolic outcomes, as a result of HIIT is warranted.

An exploratory aim of this study was to evaluate the potential modulatory effect of sex on adaptations to HIIT. Minimal differences in response between men and women were observed in this study. Analysis of change scores showed significant responses predominately occurred in men. However, in women, significant increases in thigh LM and VL mCSA were observed with HIIT + EAA after 4 weeks, with further increases in VL mCSA after 8 weeks. There is considerable debate as to whether males and females respond differently to HIIT (Forbes et al., 2020). Differences in response to moderate continuous exercise between men and women, typically favoring more positive responses in men, are predominately attributed to differences in sex hormones (Sokoloff et al., 2016; Tarnopolsky, 2008). However, recent evidence in mice suggests that HIIT may overcome these differences, promoting positive changes in both men and women (McMullan et al., 2018). Although few studies in humans have directly evaluated sex differences in response to HIIT, a majority of studies also report no effect of sex on responses to HIIT (Astorino et al., 2011; Astorino & Schubert, 2018; Bagley et al., 2016; Gillen et al., 2014; Metcalfe et al., 2012, 2016; Richards et al., 2010; Scalzo et al., 2014; Skelly et al., 2017). Specific to this study, following 12 weeks of sprint‐interval training (SIT), Heydari et al. (2012) reported significant increases in total body fat‐free mass (1.2 kg) and increased LM in the legs and trunk in overweight men (Heydari et al., 2013). After 6 weeks of SIT, Gillen et al. (2013) reported a nonsignificant 0.6 kg average increase in total body LM in overweight and obese women. Although nonsignificant, if extrapolated out to 12 weeks, this gain in LM would be equivalent to the increase reported by Heydari et al. in men. Gillen et al. (2013) also reported a significant increase in leg LM (+0.4 kg). Scalzo et al. (2014) reported greater muscle protein synthesis and mitochondrial biogenesis in men compared to women following sprint‐intervals, but no differences in oxygen consumption, time‐trial performance, or power output were reported (Scalzo et al., 2014). Although responses tended to be greater in men compared to women in this study, the addition of EAA to HIIT appeared to support greater increases in women than HIIT alone.

In conclusion, significant increases in thigh LM and improved muscle quality can be achieved with 8 weeks of HIIT training in overweight and obese adults. Supplementing HIIT with twice daily EAA supplementation may support greater increases in LM over time by increasing whole‐body protein turnover. Increases in thigh LM, mCSA, MV, and improved muscle quality can occur in as early as 4 weeks, adding to the growing body of evidence supporting the unique benefits of HIIT as a time‐efficient and effective approach for improving health outcomes (Gibala et al., 2012). Benefits appear to extend to both men and women, with EAA potentially being especially important for supporting muscular changes in women. When considering the significant improvements in cardiorespiratory fitness that are characteristic of HIIT, HIIT and EAA have significant potential for being an effective approach to improving all‐around cardiometabolic health. Compared to more traditional approaches to exercise, HIIT also requires minimal training time and lifestyle changes, warranting future investigation in more at‐risk, clinical populations.

## CONFLICT OF INTEREST

KRH, CEG, HES, LMG, CHH, GJB, MNMB, KMH, EJMD, EDR, and AESR have no conflict of interest to declare. AAF is listed as an inventor on United States patent 9,364,463 B2 entitled “Use of amino acid supplementation for improved muscle recovery,” and United States patent application 20,200,253,908 entitled “Use of amino acid supplementation for improved muscle protein synthesis.” Authors’ Contributions: KRH and AESR conceived the idea and planned the original experiments. KRH, CEG, HES, LMG, CHH, GJB, and MNMB performed the experiments. KRH compiled the results and completed analyses and also lead in writing the manuscript. AAF, KMH, EJMD, and EDR provided critical feedback that helped shape the research design, experiments, analysis, and manuscript. All authors (KRH, CEG, HES, LMG, CHH, GJB, MNMB, AAF, KMH, EJMD, EDR, AESR) provided feedback on the final manuscript.

## References

[phy214655-bib-0001] Ahtiainen, J. P. , Hoffren, M. , Hulmi, J. J. , Pietikäinen, M. , Mero, A. A. , Avela, J. , & Häkkinen, K. (2010). Panoramic ultrasonography is a valid method to measure changes in skeletal muscle cross‐sectional area. European Journal of Applied Physiology, 108(2), 273. 19777252 10.1007/s00421-009-1211-6

[phy214655-bib-0002] Areta, J. L. , Burke, L. M. , Ross, M. L. , Camera, D. M. , West, D. W. , Broad, E. M. , Jeacocke, N. A. , Moore, D. R. , Stellingwerff, T. , Phillips, S. M. , & Hawley, J. A. (2013). Timing and distribution of protein ingestion during prolonged recovery from resistance exercise alters myofibrillar protein synthesis. Journal of Physiology, 591(9), 2319–2331. 10.1113/jphysiol.2012.244897 PMC3650697 23459753

[phy214655-bib-0003] Astorino, T. A. , Allen, R. P. , Roberson, D. W. , Jurancich, M. , Lewis, R. , McCarthy, K. , & Trost, E. (2011). Adaptations to high‐intensity training are independent of gender. European Journal of Applied Physiology, 111(7), 1279–1286. 10.1007/s00421-010-1741-y 21132441

[phy214655-bib-0004] Astorino, T. A. , & Schubert, M. M. (2018). Changes in fat oxidation in response to various regimes of high intensity interval training (HIIT). European Journal of Applied Physiology, 118(1), 51–63. 10.1007/s00421-017-3756-0 29124325

[phy214655-bib-0005] Bagley, L. , Slevin, M. , Bradburn, S. , Liu, D. , Murgatroyd, C. , Morrissey, G. , Carroll, M. , Piasecki, M. , Gilmore, W. S. , & McPhee, J. S. (2016). Sex differences in the effects of 12 weeks sprint interval training on body fat mass and the rates of fatty acid oxidation and VO2max during exercise. BMJ Open Sport & Exercise Medicine, 2(1), e000056. 10.1136/bmjsem-2015-000056 PMC5117024 27900150

[phy214655-bib-0006] Blackwell, J. , Doleman, B. , Boereboom, C. L. , Morton, A. , Williams, S. , Atherton, P. , Smith, K. , Williams, J. P. , Phillips, B. E. , & Lund, J. N. (2020). High‐intensity interval training produces a significant improvement in fitness in less than 31 days before surgery for urological cancer: A randomised control trial. Prostate Cancer and Prostatic Diseases, 1–9. 10.1038/s41391-020-0219-1 PMC7655502 32157250

[phy214655-bib-0007] Blue, M. N. M. , Smith‐Ryan, A. E. , Trexler, E. T. , & Hirsch, K. R. (2017). The effects of high intensity interval training on muscle size and quality in overweight and obese adults. Journal of Science and Medicine in Sport. 10.1016/j.jsams.2017.06.001 PMC7104622 28647284

[phy214655-bib-0008] Borsheim, E. ,. Bui, Q. U. , Tissier, S. , Kobayashi, H. , Ferrando, A. A. , & Wolfe, R. R. (2008). Effect of amino acid supplementation on muscle mass, strength and physical function in elderly. Clinical Nutrition, 27(2), 189–195. 18294740 10.1016/j.clnu.2008.01.001 PMC2430042

[phy214655-bib-0009] Borsheim, E. , Tipton, K. D. , Wolf, S. E. , & Wolfe, R. R. (2002). Essential amino acids and muscle protein recovery from resistance exercise. American Journal of Physiology Endocrinology and Metabolism, 283(4), E648–E657. 12217881 10.1152/ajpendo.00466.2001

[phy214655-bib-0010] Corcoran, M. P. , Lamon‐Fava, S. , & Fielding, R. A. (2007). Skeletal muscle lipid deposition and insulin resistance: Effect of dietary fatty acids and exercise. The American Journal of Clinical Nutrition, 85(3), 662–677. 17344486 10.1093/ajcn/85.3.662

[phy214655-bib-0011] Fern, E. B. , Garlick, P. J. , & Waterlow, J. C. (1985). Apparent compartmentation of body nitrogen in one human subject: Its consequences in measuring the rate of whole‐body protein synthesis with 15N. Clinical Science (Lond), 68(3), 271–282. 10.1042/cs0680271 3918824

[phy214655-bib-0012] Ferrando, A. A. , Lane, H. W. , Stuart, C. A. , Davis‐Street, J. , & Wolfe, R. R. . (1996). Prolonged bed rest decreases skeletal muscle and whole body protein synthesis. American Journal of Physiology, 270(4 Pt 1), E627–E633. 10.1152/ajpendo.1996.270.4.E627 8928769

[phy214655-bib-0013] Forbes, S. C. , Candow, D. G. , Smith‐Ryan, A. E. , Hirsch, K. R. , Roberts, M. D. , VanDusseldorp, T. A. , Stratton, M. T. , Kaviani, M. , & Little, J. P. (2020). Supplements and Nutritional Interventions to Augment High‐Intensity Interval Training Physiological and Performance Adaptations—A Narrative Review. Nutrients, 12(2), 390. 10.3390/nu12020390 PMC7071320 32024038

[phy214655-bib-0014] Fothergill, E. , Guo, J. , Howard, L. , Kerns, J. C. , Knuth, N. D. , Brychta, R. , Chen, K. Y. , Skarulis, M. C. , Walter, M. , Walter, P. J. , & Hall, K. D. (2016). Persistent metabolic adaptation 6 years after "The Biggest Loser" competition. Obesity (Silver Spring), 24(8), 1612–1619. 10.1002/oby.21538 27136388 PMC4989512

[phy214655-bib-0015] Franchi, M. V. , Atherton, P. J. , Reeves, N. D. , Flück, M. , Williams, J. , Mitchell, W. K. , Selby, A. , Beltran Valls, R. M. , & Narici, M. V. (2014). Architectural, functional and molecular responses to concentric and eccentric loading in human skeletal muscle. Acta Physiologica, 210(3), 642–654. 24387247 10.1111/apha.12225

[phy214655-bib-0016] Gerstner, G. R. , Thompson, B. J. , Rosenberg, J. G. , Sobolewski, E. J. , Scharville, M. J. , & Ryan, E. D. (2017). Neural and Muscular Contributions to the Age‐Related Reductions in Rapid Strength. Medicine and Science in Sports and Exercise, 49(7), 1331–1339. 10.1249/MSS.0000000000001231 28166121

[phy214655-bib-0017] Gibala, M. J. , Little, J. P. , MacDonald, M. J. , & Hawley, J. A. (2012). Physiological adaptations to low‐volume, high‐intensity interval training in health and disease. The Journal of Physiology, 590(5), 1077–1084. 10.1113/jphysiol.2011.224725 22289907 PMC3381816

[phy214655-bib-0018] Gillen, J. B. , Percival, M. E. , Ludzki, A. , Tarnopolsky, M. A. , & Gibala, M. J. (2013). Interval training in the fed or fasted state improves body composition and muscle oxidative capacity in overweight women. Obesity, 21(11), 2249–2255. 23723099 10.1002/oby.20379

[phy214655-bib-0019] Gillen, J. B. , Percival, M. E. , Skelly, L. E. , Martin, B. J. , Tan, R. B. , Tarnopolsky, M. A. , & Gibala, M. J. (2014). Three minutes of all‐out intermittent exercise per week increases skeletal muscle oxidative capacity and improves cardiometabolic health. PLoS One, 9(11), e111489 10.1371/journal.pone.0111489 25365337 PMC4218754

[phy214655-bib-0020] Goodpaster, B. H. ,. Park, S. W. , Harris, T. B. , Kritchevsky, S. B. , Nevitt, M. , Schwartz, A. V. , Simonsick, E. M. , Tylavsky, F. A. , Visser, M. , & Newman, A. B. (2006). The loss of skeletal muscle strength, mass, and quality in older adults: The health, aging and body composition study. The Journals of Gerontology Series A: Biological Sciences and Medical Sciences, 61(10), 1059–1064. 10.1093/gerona/61.10.1059 17077199

[phy214655-bib-0021] Haun, C. T. , Vann, C. G. , Osburn, S. C. , Mumford, P. W. , Roberson, P. A. , Romero, M. A. , Fox, C. D. , Johnson, C. A. , Parry, H. A. , Kavazis, A. N. , & Moon, J. R. (2019). Muscle fiber hypertrophy in response to 6 weeks of high‐volume resistance training in trained young men is largely attributed to sarcoplasmic hypertrophy. PLoS One, 14(6). 10.1371/journal.pone.0215267 PMC6550381 31166954

[phy214655-bib-0022] Heydari, M. , Boutcher, Y. N. , & Boutcher, S. H. (2013). The effects of high‐intensity intermittent exercise training on cardiovascular response to mental and physical challenge. International Journal of Psychophysiology : Official Journal of the International Organization of Psychophysiology, 87(2), 141–146. 10.1016/j.ijpsycho.2012.11.013 23220158

[phy214655-bib-0023] Heydari, M. , Freund, J. , & Boutcher, S. H. (2012). The effect of high‐intensity intermittent exercise on body composition of overweight young males. Journal of Obesity, 2012, 480467. 22720138 10.1155/2012/480467 PMC3375095

[phy214655-bib-0024] Hirsch, K.R. , Greenwalt, C.E. , Saylor, H.E. , Gould, L.M. , Brewer, G.J. , Blue, M.N.M. , Ferrando, M.N.M. , Huffman, K.M. , Mayer‐David, E.J. , Ryan, E.D. , Smith‐Ryan, A.E. Metabolic effects of high‐intensity interval training and essential amino acids. In Review, 2020. 10.1007/s00421-021-04792-4 34427732

[phy214655-bib-0025] Hirsch, K. R. , Smith‐Ryan, A. E. , Blue, M. N. , Mock, M. G. , & Trexler, E. T. (2017). Influence of segmental body composition and adiposity hormones on resting metabolic rate and substrate utilization in overweight and obese adults. Journal of Endocrinological Investigation, 40(6), 635–643. 10.1007/s40618-017-0616-z 28211029 PMC5444984

[phy214655-bib-0026] Hirsch, K. R. , Smith‐Ryan, A. E. , Trexler, E. T. , & Roelofs, E. J. (2016). Body composition and muscle characteristics of division I track and field athletes. Journal of Strength and Conditioning Research, 30(5), 1231–1238. 10.1519/JSC.0000000000001203 27100166 PMC4843842

[phy214655-bib-0027] Hogrel, J. Y. , Barnouin, Y. , Azzabou, N. , Butler‐Browne, G. , Voit, T. , Moraux, A. , Leroux, G. , Behin, A. , McPhee, J. S. , & Carlier, P. G. (2015). NMR imaging estimates of muscle volume and intramuscular fat infiltration in the thigh: Variations with muscle, gender, and age. Age, 37(3), 9798 10.1007/s11357-015-9798-5 26040416 PMC4456487

[phy214655-bib-0028] Hulmi, J. J. , Lockwood, C. M. , & Stout, J. R. (2010). Effect of protein/essential amino acids and resistance training on skeletal muscle hypertrophy: A case for whey protein. Nutrition & Metabolism, 7, 51. 20565767 10.1186/1743-7075-7-51 PMC2901380

[phy214655-bib-0029] Jager, R. , Kerksick, C. M. , Campbell, B. I. , Cribb, P. J. , Wells, S. D. , Skwiat, T. M. , Purpura, M. , Ziegenfuss, T. N. , Ferrando, A. A. , Arent, S. M. , & Smith‐Ryan, A. E. (2017). International Society of Sports Nutrition Position Stand: Protein and exercise. Journal of the International Society of Sports Nutrition, 14, 20. 28642676 10.1186/s12970-017-0177-8 PMC5477153

[phy214655-bib-0030] Janssen, I. , Heymsfield, S. B. , Wang, Z. , & Ross, R. (2000). Skeletal muscle mass and distribution in 468 men and women aged 18–88 yr. Journal of Applied Physiology, 89(1), 81–88. 10.1152/jappl.2000.89.1.81 10904038

[phy214655-bib-0031] Macpherson, R. E. , Hazell, T. J. , Olver, T. D. , Paterson, D. H. , & Lemon, P. W. R. (2011). Run sprint interval training improves aerobic performance but not maximal cardiac output. Medicine and Science in Sports and Exercise, 43(1), 115–122. 20473222 10.1249/MSS.0b013e3181e5eacd

[phy214655-bib-0032] Mangine, G. T. , Redd, M. J. , Gonzalez, A. M. , Townsend, J. R. , Wells, A. J. , Jajtner, A. R. , Beyer, K. S. , Boone, C. H. , La Monica, M. B. , Stout, J. R. , & Fukuda, D. H. (2018). Resistance training does not induce uniform adaptations to quadriceps. PLoS One, 13(8). 10.1371/journal.pone.0198304 PMC6116919 30161137

[phy214655-bib-0033] McMullan, R. C. , Ferris, M. T. , Bell, T. A. , Menachery, V. D. , Baric, R. S. , Hua, K. , Pomp, D. , SmithRyan, A. E. , & de Villena, F. P. (2018). CC 002/Unc females are mouse models of exercise‐induced paradoxical fat response. Physiological Reports, 6(12), e13716. 29924460 10.14814/phy2.13716 PMC6009762

[phy214655-bib-0034] Melvin, M. N. , Smith‐Ryan, A. E. , Wingfield, H. L. , Ryan, E. D. , Trexler, E. T. , & Roelofs, E. J. (2014). Muscle characteristics and body composition of NCAA division I football players. Journal of Strength and Conditioning Research, 28(12), 3320–3329. 10.1519/JSC.0000000000000651 25187245

[phy214655-bib-0035] Metcalfe, R. S. , Babraj, J. A. , Fawkner, S. G. , & Vollaard, N. B. (2012). Towards the minimal amount of exercise for improving metabolic health: Beneficial effects of reduced‐exertion high‐intensity interval training. European Journal of Applied Physiology, 112(7), 2767–2775. 10.1007/s00421-011-2254-z 22124524

[phy214655-bib-0036] Metcalfe, R. S. , Tardif, N. , Thompson, D. , & Vollaard, N. B. (2016). Changes in aerobic capacity and glycaemic control in response to reduced‐exertion high‐intensity interval training (REHIT) are not different between sedentary men and women. Applied Physiology, Nutrition, and Metabolism Physiologie Appliquee, Nutrition Et Metabolisme, 41(11), 1117–1123. 10.1139/apnm-2016-0253 27753506

[phy214655-bib-0037] Miller, S. L. , Tipton, K. D. , Chinkes, D. L. , Wolf, S. E. , & Wolfe, R. R. (2003). Independent and combined effects of amino acids and glucose after resistance exercise. Medicine and Science in Sports and Exercise, 35(3), 449–455. 12618575 10.1249/01.MSS.0000053910.63105.45

[phy214655-bib-0038] Moghaddam, M. , Estrada, C. A. , Baghurst, T. , & Jacobson, B. H. (2020). Muscular morphological adaptations of two whole‐body high intensity interval training (HIIT) configurations. The Journal of Sports Medicine and Physical Fitness. 10.23736/S0022-4707.20.10526-7 32343081

[phy214655-bib-0039] Morse, C. I. , Degens, H. , & Jones, D. A. (2007). The validity of estimating quadriceps volume from single MRI cross‐sections in young men. European Journal of Applied Physiology, 100(3), 267–274. 10.1007/s00421-007-0429-4 17342544

[phy214655-bib-0040] Mota, J. A. , Stock, M. S. , & Thompson, B. J. (2017). Vastus lateralis and rectus femoris echo intensity fail to reflect knee extensor specific tension in middle‐school boys. Physiological Measurement, 38(8), 1529–1541. 10.1088/1361-6579/aa791a 28607221

[phy214655-bib-0041] Narici, M. , Franchi, M. , & Maganaris, C. (2016). Muscle structural assembly and functional consequences. Journal of Experimental Biology, 219(2), 276–284. 10.1242/jeb.128017 26792340

[phy214655-bib-0042] Rasmussen, B. B. , Tipton, K. D. , Miller, S. L. , Wolf, S. E. , & Wolfe, R. R. (2000). An oral essential amino acid‐carbohydrate supplement enhances muscle protein anabolism after resistance exercise. Journal of Applied Physiology, 88(2), 386–392. 10658002 10.1152/jappl.2000.88.2.386

[phy214655-bib-0043] Rech, A. , Radaelli, R. , Goltz, F. R. , da Rosa, L. H. , Schneider, C. D. , & Pinto, R. S. (2014). Echo intensity is negatively associated with functional capacity in older women. Age, 36(5), 9708 10.1007/s11357-014-9708-2 25167965 PMC4453939

[phy214655-bib-0044] Richards, J. C. , Johnson, T. K. , Kuzma, J. N. , Lonac, M. C. , Schweder, M. M. , Voyles, W. F. , & Bell, C. (2010). Short‐term sprint interval training increases insulin sensitivity in healthy adults but does not affect the thermogenic response to β‐adrenergic stimulation. The Journal of Physiology, 588(15), 2961–2972. 10.1113/jphysiol.2010.189886 20547683 PMC2956910

[phy214655-bib-0045] Scalzo, R. L. , Peltonen, G. L. , Binns, S. E. , Shankaran, M. , Giordano, G. R. , Hartley, D. A. , Klochak, A. L. , Lonac, M. C. , Paris, H. L. , Szallar, S. E. , & Wood, L. M. (2014). Greater muscle protein synthesis and mitochondrial biogenesis in males compared with females during sprint interval training. The FASEB Journal, 28(6), 2705–2714. 24599968 10.1096/fj.13-246595

[phy214655-bib-0046] Shah, N. R. , & Braverman, E. R. (2012). Measuring adiposity in patients: The utility of body mass index (BMI), percent body fat, and leptin. PLoS One, 7(4), e33308. 22485140 10.1371/journal.pone.0033308 PMC3317663

[phy214655-bib-0047] Skelly, L. E. , Gillen, J. B. , MacInnis, M. J. , Martin, B. J. , Safdar, A. , Akhtar, M. , MacDonald, M. J. , Tarnopolsky, M. A. , & Gibala, M. J. (2017). Effect of sex on the acute skeletal muscle response to sprint interval exercise. Experimental Physiology, 102(3), 354–365. 10.1113/EP086118 28118678

[phy214655-bib-0048] Smith‐Ryan, A. E. , Melvin, M. N. , & Wingfield, H. L. (2015). High‐intensity interval training: Modulating interval duration in overweight/obese men. Phys Sportsmed, 43(2), 107–113. 25913937 10.1080/00913847.2015.1037231 PMC4427241

[phy214655-bib-0049] Smith‐Ryan, A. E. , Trexler, E. T. , Wingfield, H. L. , & Blue, M. N. (2016). Effects of high‐intensity interval training on cardiometabolic risk factors in overweight/obese women. Journal of Sports Sciences, 34(21), 2038–2046. 26934687 10.1080/02640414.2016.1149609 PMC5010533

[phy214655-bib-0050] Sokoloff, N. C. , Misra, M. , & Ackerman, K. E. (2016). Exercise, training, and the hypothalamic‐pituitary‐gonadal axis in men and women. In Sports Endocrinology Karger Publishers 47, p. 27–43. 10.1159/000445154 PMC7043068 27348623

[phy214655-bib-0051] Stein, T. P. , Rumpler, W. V. , Leskiw, M. J. , Schluter, M. D. , Staples, R. , & Bodwell, C. E. (1991). Effect of reduced dietary intake on energy expenditure, protein turnover, and glucose cycling in man. Metabolism, 40(5), 478–483. 10.1016/0026-0495(91)90228-O 2023534

[phy214655-bib-0052] Tarnopolsky, M. A. (2008). Sex differences in exercise metabolism and the role of 17‐beta estradiol. Medicine and Science in Sports and Exercise, 40(4), 648–654. 18317381 10.1249/MSS.0b013e31816212ff

[phy214655-bib-0053] Tipton, K. D. et al (2001). Timing of amino acid‐carbohydrate ingestion alters anabolic response of muscle to resistance exercise. American journal of physiology Endocrinology and Metabolism, 281(2), E197–206. 11440894 10.1152/ajpendo.2001.281.2.E197

[phy214655-bib-0054] Tipton, K. D. , Rasmussen, B. B. , Miller, S. L. , Wolf, S. E. , Owens‐Stovall, S. K. , Petrini, B. E. , & Wolfe, R. R. (2001). Timing of amino acid‐carbohydrate ingestion alters anabolic response of muscle to resistance exercise. American Journal of Physiology. Endocrinology and Metabolism, 281(2), E197–206. 11440894 10.1152/ajpendo.2001.281.2.E197

[phy214655-bib-0055] Wewege, M. , Van Den Berg, R. , Ward, R. E. , & Keech, A. (2017). The effects of high‐intensity interval training vs. moderate‐intensity continuous training on body composition in overweight and obese adults: A systematic review and meta‐analysis. Obesity Reviews, 18(6), 635–646. 10.1111/obr.12532 28401638

[phy214655-bib-0056] Wolfe, R. R. (2001). Effects of amino acid intake on anabolic processes. Canadian Journal of Applied Physiology, 26(S1), S220–S227. 11897897 10.1139/h2001-056

[phy214655-bib-0057] Wolfe, R. R. (2006). The underappreciated role of muscle in health and disease. American Journal of Clinical Nutrition, 84(3), 475–482. 10.1093/ajcn/84.3.475 16960159

[phy214655-bib-0058] Young, H. J. , Jenkins, N. T. , Zhao, Q. , & McCully, K. K. (2015). Measurement of intramuscular fat by muscle echo intensity. Muscle and Nerve, 52(6), 963–971. 25787260 10.1002/mus.24656 PMC4575231

